# Climax forest has a higher soil bacterial diversity but lower soil nutrient contents than degraded forests in temperate northern China

**DOI:** 10.1002/ece3.9535

**Published:** 2022-11-22

**Authors:** Xin Sui, Mengsha Li, Beat Frey, Mingyu Wang, Xiaohong Weng, Xin Wang, Fuyuan Chen, Xianda Li, Zhong Du, Libin Yang, Mai‐He Li

**Affiliations:** ^1^ Engineering Research Center of Agricultural Microbiology Technology, Ministry of Education & Heilongjiang Provincial Key Laboratory of Ecological Restoration and Resource Utilization for Cold Region & Key Laboratory of Microbiology, College of Heilongjiang Province & School of Life Sciences Heilongjiang University Harbin China; ^2^ Swiss Federal Institute for Forest, Snow and Landscape Research WSL Birmensdorf Switzerland; ^3^ Institute of Nature and Ecology Heilongjiang Academy of Sciences Harbin China; ^4^ Heilongjiang Zhongyangzhan Black‐Billed Capercaillie Nature Reserve Administration Bureau Nenjiang China; ^5^ School of Geographical Sciences West Normal University Nanchong China; ^6^ Key Laboratory of Geographical Processes and Ecological Security in Changbai Mountains, Ministry of Education, School of Geographical Sciences Northeast Normal University Changchun China; ^7^ School of Life Science Hebei University Baoding China

**Keywords:** amplicon sequencing, bacterial diversity, forest soil, forest vegetation type, soil bacteria

## Abstract

Bacteria are a crucial component of forest soil biodiversity and play an important role in numerous ecosystem processes. Here, we studied the patterns of soil bacterial community diversity and structure in a climax forest (*Larix gmelinii*; LG) compared with those in degraded forest ecosystems of four forest vegetation types (BD, *Betula dahurica*; BP, *Betula platyphylla*; QM, *Quercus mongolica*; and LGQM, a mixed coniferous–broadleaved forest composed of *Larix gmelinii* and *Quercus mongolica*) in the Heilongjiang Zhongyangzhan Black‐billed Capercaillie Nature Reserve in northern China, using Illumina MiSeq sequencing of 16 S rRNA genes. Soil physicochemical properties (pH, soil organic carbon = SOC, total nitrogen = TN, carbon/nitrogen = C/N, total phosphorous = TP, available nitrogen = AN, available phosphorous = AP) differed significantly (*p* < .05) among the five forests. SOC, C/N, TP, AN, and AP were highest in QM, whereas SOC was lowest in LGQM. Soil pH was lowest in BD and highest in LGQM. α diversity was highest in LG and lowest in QM. The soil bacterial community composition in the climax forest was significantly different from that in the four degraded forests (*p* < .05). The dominant bacterial phyla were Acidobacteria, Proteobacteria, Verrucomicrobia, Bacteroidetes, Actinobacteria, Gemmatimonadetes, Firmicutes, Chloroflexi, and Rokubacteria. The highest relative abundances of these phyla were: 30.7% for Acidobacteria in LGQM, 42.6% for Proteobacteria in LG, 17.6% for Verrucomicrobia in BD, 5.5% for Firmicutes in BP, and 6.9% for Actinobacteria in QM. The dominant bacterial genera across the five forest vegetation types were *Bryobacter* and some poorly characterized taxa (e.g., *Candidatus_Udaeobacter* and *Candidatus_Solibacter*). Redundancy analysis indicated that SOC, C/N, TP, AN, and AP were the main soil physicochemical properties that shaped the bacterial communities. Our study revealed distinct bacterial diversity and composition in the climax forest compared with values in degraded forests, suggesting that the biotic and abiotic factors associated with climax ecosystems play an important role in shaping soil bacterial community structure and thus biogeochemical functions. The results of this study contribute to a deeper understanding and better predictions of the network among belowground systems and of the functions and services of degraded forests compared with climax ecosystems.

## Graphical abstract

Soil bacterial diversities of conifer forests were significantly higher than broad‐leaved forests as well as mixed coniferous and broad‐leaved forests. Soil organic, C/N, total phosphorus, available phosphorus and available nitrogen were the main drivers of soil diversity and community structure.

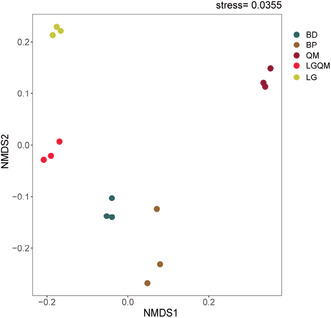

## 1 INTRODUCTION

Forests are the main type of terrestrial ecosystem, and they play a crucial role in maintaining biodiversity and the global climate (Deng, Zhou, et al., 2019; Sui et al., 2022). Soil microorganisms are a crucial component of forests (Adamczyk et al., 2019) and are involved in litter decomposition and biogeochemical cycling (Kögel‐Knabner, 2002; Pisani et al., 2015; Widdig et al., 2020), thus playing an important role in maintaining forest ecosystem structure, function, and stability (Chai et al., 2019; Qiang et al., 2021). On the other hand, changes in forest vegetation type lead to changes in soil microbial structure, function, and diversity (Deng, Zhou, et al., 2019; Frey et al., 2021). There is a co‐evolutionary relationship between plants and soil microorganisms. Plants provide nutrient substrates to soil microorganisms, while soil microorganisms decompose more than 90% of all plant litter, converting organic matter into inorganic nutrients required for plant growth. Understanding changes in microbial communities and the main factors driving these changes can shed light on the mechanisms behind the impacts of climate change, natural disturbances, and human activities on the structure, function, and stability of forest ecosystems.

Many previous studies have demonstrated that changes in soil properties, plant diversity, and community structure can affect the composition, function, and diversity of soil bacteria (Chai et al., 2019; Qiang et al., 2021). Vegetation type has been reported to be an essential factor affecting soil bacterial communities, given the same climatic conditions (Deng, Zhang, et al., 2019). Specifically, the quality and quantity of plant litter and root exudates can differ considerably among vegetation types, which in turn affects the structure of soil bacterial communities (Bach et al., 2010). However, studies have produced inconsistent results regarding soil bacterial structure and diversity in relation to forest vegetation type. For instance, the abundance of soil bacteria has been found to be much higher in broadleaved forest soils than in coniferous forest soils (Xia et al., 2012), and soil bacterial community structure and species diversity have been reported to vary significantly with forest type (Lin et al., 2021). In contrast, Chen et al. (2022) found that the soil bacterial Shannon diversity index and richness did not differ significantly among four tropical rainforests, although community structure did differ. Moreover, in a study in the Chinese Daxing'anling mountains, Yang et al. (2018) found that the soil bacterial Shannon index and richness changed significantly in three coniferous forests dominated by *Larix gmelinii* but with different understory species. The variation in these results may be caused by various biotic and abiotic factors, as well as their interactions, in different forest vegetation types. For example, the biotic and abiotic factors in a climax ecosystem differ greatly from those in ecosystems in earlier successional stages (Sui et al., 2021). However, the changes in the soil bacteria community that occur with succession have rarely been studied. In this study, we, therefore, considered the soil bacterial community in a *L. gmelinii* climax forest compared with those in degraded forests in temperate northern China.

*Larix gmelinii* is the climax forest ecosystem in the Heilongjiang Zhongyangzhan Black‐billed Capercaillie Nature Reserve, located in the Daxing'anling and Xiaoxing'anling mountains in temperate northern China. However, due to excessive logging and other disturbances, the *L. gmelinii* forests have been seriously degraded into various other vegetation types, including coniferous–broadleaved mixed forests and broadleaved forests. The effects of these degradations on the soil bacterial community have never been investigated. The intermediate disturbance hypothesis states that the species diversity is highest when an ecosystem is moderately disturbed (Fox, 1979), and thus, degraded forests may have higher soil microbial diversity than a climax forest. Therefore, we investigated the soil bacterial community in four degraded forest vegetation types (i.e., *Betula dahurica* forests, *Betula platyphylla* forests, *Quercus mongolica* forests, and *Larix gmelinii* and *Quercus mongolica* mixed forests) compared with that in the climax (*Larix gmelinii*) forest ecosystem in that region. We measured soil bacterial composition, diversity, and function using Illumina MiSeq sequencing of the 16 S rRNA genes (V3–V4). We hypothesized that: (1) the degraded forests would have better soil conditions than the climax *L. gmelinii* forest, owing to the occurrence of broadleaved trees in the degraded forests, and thus (2) the soil bacterial community in the degraded forests would have a higher diversity and a more complex structure than the climax forest. The overall aim of this study was to contribute to a deeper understanding and better predictions of the network among belowground systems and of the functions and services of climax ecosystems.

## 2 MATERIAL AND METHODS

### 2.1 Experimental site

**FIGURE A1:**
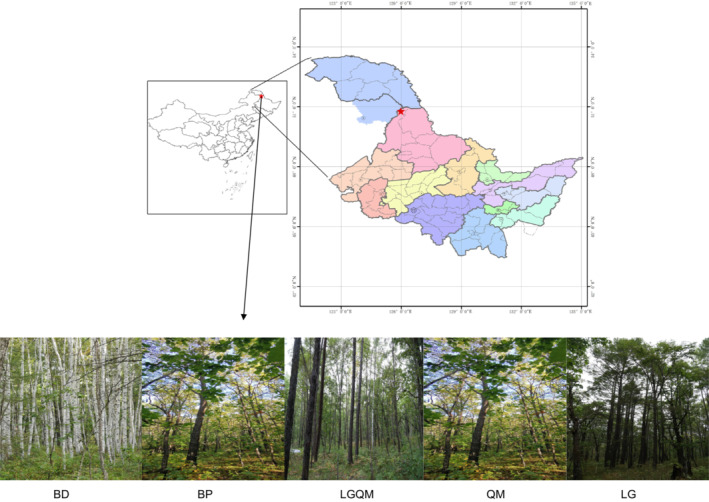
Study site location in northeastern China and images of the five forests. BD, *Betula dahurica* forest; BP, *Betula platyphylla* forest; LG, *Larix gmelinii* forest; LGQM, *Q. mongolica* and *L. gmelinii* mixed forest; QM, *Quercus mongolica* forest.

The experimental site was located in the Heilongjiang Zhongyangzhan Black‐billed Capercaillie Nature Reserve (126°00′–126°45′N, 48°30′–48°50′E; Figure A1 in Appendix), Heilongjiang Province, China. The nature reserve covers a total area of 46,743 ha and belongs to the staggered area of the Daxing'anling and Xiaoxing'anling mountain system. This area is characterized by a cold temperate climate, with long cold winters, short warm and wet summers, and higher diurnal temperature variation. The mean annual temperature is −0.4°C, and the mean annual precipitation is 450–550 mm. The primitive conifer (*Larix gmelinii*, LG) forest and the following four secondary forests were selected for our research: *Betula dahurica* forests (BD), *Betula platyphylla* forests (BP), *Quercus mongolica* forests (QM), and *Larix gmelinii* and *Quercus mongolica* mixed forests (LGQM). All forests are older than 40 years. The soil is deeper than 25 cm, developed from granite and classified as Borice Luvisols, according to the American Soil Taxonomy. Table A1 in Appendix summarizes the characteristics of the five forests.

### 2.2 Soil sampling

In July 2019, soil samples were collected from the five forests. Three independent plots (20 m × 20 m each) were established in each forest. Fifteen to 20 soil samples (0–20 cm) were obtained, using a soil auger (5 cm in diameter, 20 cm deep) after removing the litter layer from each plot. Approximately 2.5 kg soil was collected from each plot. The soil samples were immediately placed in an icebox and kept at 4°C until processing in the laboratory. The soil samples were then pooled to form a single composite soil sample per plot and passed through a 2‐mm mesh to remove the roots and other debris. Each composite sample was then divided into two sub‐samples; one was stored at −80°C for DNA extraction (approximately 10 g soil), whereas the other was air‐dried for soil physicochemical analyses (approximately 1 kg soil).

### 2.3 Soil physicochemical properties

The methods used to measure soil pH, soil organic carbon (SOC), total nitrogen (TN), available nitrogen (AN), total phosphorous (TP), and available phosphorus (AP) were described by Sui et al. (2022). Briefly, soil pH was measured using a pH meter and a soil to water ratio of 1:2.5 w/v. SOC and TN were measured using an elemental analyzer (Elementar). AN was measured using a continuous flow analysis system (SKALAR SAN++). TP was measured using a spectrophotometer, and AP was measured using the colorimetric method upon extraction with 0.5 M NaHCO_3_.

### 2.4 Soil DNA extraction and 16 S rDNA sequencing

Soil total DNA was extracted using the Power Soil DNA isolation kit 12888 according to the manufacturer's instructions. Using 50 ng DNA as a template, the 338F (5′‐ACT CCT ACG GGA GGC AGC A‐3′) and 806R (5′‐GGA CTA CHV GGG TWT CTA AT‐3′) universal primers were used to amplify the V3–V4 regions (Liu et al., 2016). The PCR amplification system consisted of 12.5 μl PCR mix, 1.2 μl forward and reverse primers (5 μmol·L^−1^), 1 μl DNA template (50 ng·L^−1^), and enough ultrapure water (ddH_2_O) to reach a 25 μl reaction volume. The amplification conditions as follows: pre‐denaturation at 95°C for 5 min, 35 cycles of denaturation at 95°C for 30 s, annealing at 52°C for 45 s, extension at 60°C for 60 s, and finally an extension step at 72°C for 10 min. The PCR products were inspected using 2% agarose electrophoresis, and the PCR amplicons were purified with the AxyPrep DNA purification kit (MAG‐PCR‐CL‐5, Axygen). Three independent PCR replicates were produced per sample then pooled in equal amounts; the pooled samples were then paired‐end sequenced on the Illumina MiSeq v3 platform (2 × 300 bp). The raw sequences were uploaded to the NCBI Sequence Read Archive database under accession number PRJNA691134.

### 2.5 Bioinformatics and statistical analysis

The QIIME2 microbiome bioinformatics platform (Oksanen, 2015) was used to process the raw FASTA sequences. Forward and reverse reads were merged using the PEAR software (v0.9.8). Sequences were removed if the mean quality score was <20 or the if the length was <200 bp, and ambiguous sequences were also removed. Chimeras were removed using Usearch (v7.1, https://drive5.com/usearch/). Exact barcode matching was implemented, which permitted a two‐nucleotide mismatch during primer matching. The obtained operational taxonomic units (OTUs) were clustered by CD–HIT at a similarity threshold of 97%, and the taxonomy was annotated to the SILVA database (v138.1). Non‐bacterial sequences (e.g., mitochondria, chloroplasts) needed to be removed from OTU tables prior to statistical analyses, which were implemented using R v3.3.2 (Oksanen, 2015). Before further analysis of α diversity, the sequences were normalized according to the lowest number of sequences for a single sample. To compare the bacterial taxa abundances in soils from the five forests, we selected the non‐normality taxa abundances. Relative abundances <1% were classified as “other”.

Differences in soil physicochemical properties between the five forests were analyzed using one‐way ANOVA at a 0.05 significance level coupled with Duncan's tests, using SPSS software v17.0 (SPSS Inc.). OTU‐level α diversity indices, such as richness, Chao1 index, Abundance‐based coverage estimator (ACE), and Shannon index, were calculated using the OTU table in QIIME2. Venn diagrams, rarefaction curves, and heatmap representations of the relative abundances of bacterial OTUs among soil samples were generated using the “vegan” package in R (Oksanen, 2015). The correlation heatmaps between the soil physicochemical properties and soil bacteria (OTUs) were generated in R using “vegan” package, and only the 50 strongest correlations were displayed in the correlation heatmap. Non‐metric multidimensional scaling (NMDS) was conducted based on the Bray–Curtis dissimilarity at the OTU level, also using “vegan” (Frey et al., 2021; Hartmann et al., 2017). Redundancy analysis (RDA), implemented in “vegan”, was used to generate compositional profiles, agglomerating the OTUs to the phylum level and genus level. Indicator species analysis based on the OTU level was performed as outlined in Rime et al. (2015) and Frey et al. (2016) using “vegan” and the “labdsv” (version 1.2‐2) R package (Roberts & Roberts, 2016). The FAPROTAX functions based on the bacterial OTU level were analyzed as described by Louca et al. (2016). The FAPROTAX code and database were downloaded at http://www.loucalab.com/archive/FAPROTAX/lib/php/index.php?section=Download.

## 3 RESULTS

### 3.1 Soil physicochemical properties

Overall there were significant differences (*p* < .05) in soil physicochemical properties among the five forests (Table 1). SOC, C/N, TP, AN, and AP were highest in QM, and SOC and TN were lowest in LGQM. Soil pH and TN were lowest in BD, while pH was highest in LGQM and TN was the highest in BP (Table 1).

**TABLE 1:** Physicochemical characteristics of the soil samples in the five forest vegetation types.

Variable	BD	BP	QM	LGQM	LG
pH	4.6 ± 0.4 b	5.3 ± 0.1 a	5.4 ± 0.2 a	5.9 ± 0.3 a	5.5 ± 0.1 a
SOC (g·kg^−1^)	54.0 ± 3.5 d	76.2 ± 2.0 b	107.4 ± 22.4 a	50.0 ± 4.0 d	60.3 ± 3.5 c
TN (g·kg^−1^)	3.5 ± 0.3 b	4.3 ± 0.2 a	4.2 ± 0.1 a	2.8 ± 0.1 c	4.1 ± 0.1 a
C/N	15.6 ± 0.8 c	17.9 ± 0.4 b	25.8 ± 0.6 a	18.1 ± 1.6 b	14.7 ± 1.3 c
TP (g·kg^−1^)	2.1 ± 0.1 bc	2.3 ± 0.2 ab	2.6 ± 0.2 a	2.2 ± 0.3 bc	1.8 ± 0.2 c
AN (mg·kg^−1^)	78.7 ± 3.0 b	83.8 ± 2.2 a	87.3 ± 1.6 a	38.6 ± 2.2 c	24.6 ± 2.6 d
AP (mg·kg^−1^)	36.2 ± 1.2 b	37.9 ± 0.4 b	41.3 ± 1.4 a	35.9 ± 1.01 b	27.4 ± 0.8 c

*Note*: All results are reported as mean ± standard deviation (*n* = 3). Different letters within a row indicate significant differences (*p* < .05; ANOVA) among the five forest types tested in this study.

Abbreviations: AN, available nitrogen; AP, available phosphorous; BD, *Betula dahurica* forest; BP, *Betula platyphylla* forest; LG, *Larix gmelinii* forest; LGQM, *Q. mongolica* and *L. gmelinii* mixed forest; QM, *Quercus mongolica* forest; SOC, soil organic carbon; TN, total nitrogen; TP, total phosphorus.

### 3.2 Rarefaction curves

**FIGURE A2:**
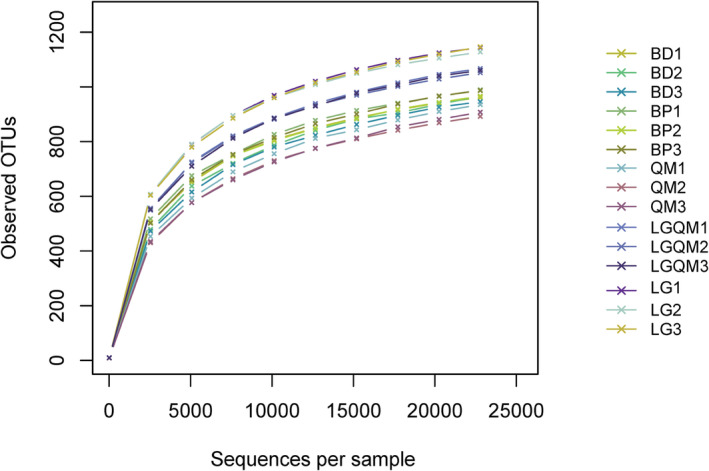
Rarefaction curves of soil samples from different forest vegetation types. BD, *Betula dahurica* forest; BP, *Betula platyphylla* forest; LG, *Larix gmelinii* forest; LGQM, *Q. mongolica* and *L. gmelinii* mixed forest; OTU, operational taxonomic unit; QM, *Quercus mongolica* forest.

The rarefaction curves of the different soil samples tended to be saturated (Figure A2 in Appendix), indicating that the data obtained from all soil samples were representative of the entire soil bacterial communities in the corresponding locations.

### 3.3 Soil α and β diversity of the soil bacterial community

After quality filtering, 828,418 sequences were retained, accounting for 2338 OTUs in total. The α diversity (i.e., Shannon index, Chao1 index, ACE, and richness) of soil bacteria differed significantly with forest type (*p* < .05; Table 1). In particular, the Shannon index, Chao1 index, ACE, and richness were highest in LG and lowest in QM (Table 2). Overall, the α diversity of the soil bacteria followed the order: coniferous forest (LG) > mixed forest (LGQM) > broadleaved forest (BD, BP, and QM).

**TABLE 2:** Bacterial α diversity of the five forest vegetation types.

Forest type	Richness	Chao1	ACE	Shannon
BD	976 ± 8 c	1108 ± 28 c	1102 ± 27 c	5.35 ± 0.1 c
BP	1000 ± 14 c	1143 ± 22 bc	1131 ± 14 c	5.59 ± 0.0 b
QM	933 ± 23 d	1088 ± 62 c	1087 ± 48 c	5.16 ± 0.0 d
LGQM	1081 ± 8 b	1201 ± 22 b	1211 ± 15 b	5.67 ± 0.0 b
LG	1160 ± 10 a	1299 ± 43 a	1284 ± 26 a	5.80 ± 0.0 a

*Note*: All results are reported as mean ± standard deviation (*n* = 3). Different letters within a row indicate significant differences (*p* < .05) among the five forests tested in this study.

Abbreviations: BD, *Betula dahurica* forest; BP, *Betula platyphylla* forest; LG, *Larix gmelinii* forest; LGQM, *Q. mongolica* and *L. gmelinii* mixed forest; QM, *Quercus mongolica* forest.

**FIGURE 1:**
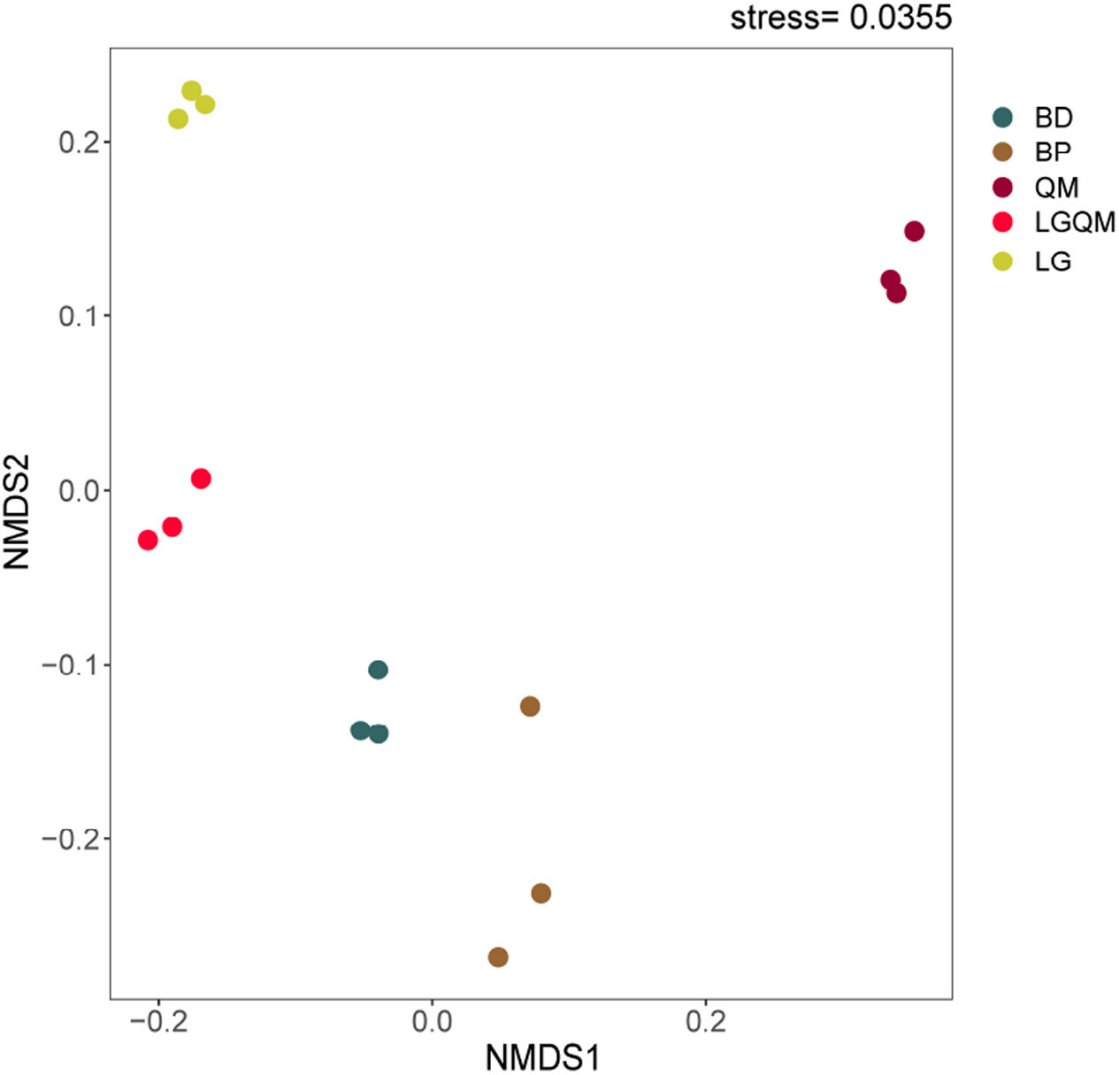
Non‐metric multidimensional scaling (NMDS) of the bacterial community structure in different forest vegetation types. BD, *Betula dahurica* forest; BP, *Betula platyphylla* forest; LG, *Larix gmelinii* forest; LGQM, *Q. mongolica* and *L. gmelinii* mixed forest; QM, *Quercus mongolica* forest.

The bacterial community structure differed significantly among the forests (PERMANOVA: *r* = .43, *p* < .01; Figure 1). The soil bacterial community structure differed significantly between coniferous forest and broadleaved forest (*p* < .05). The bacterial community structure of the two *Betula* forests (BD and BP) was similar, whereas the community structure of QM was significantly different from that of the four other forests (Figure 1).

### 3.4 Soil bacterial community composition in the five forests

**FIGURE 2:**
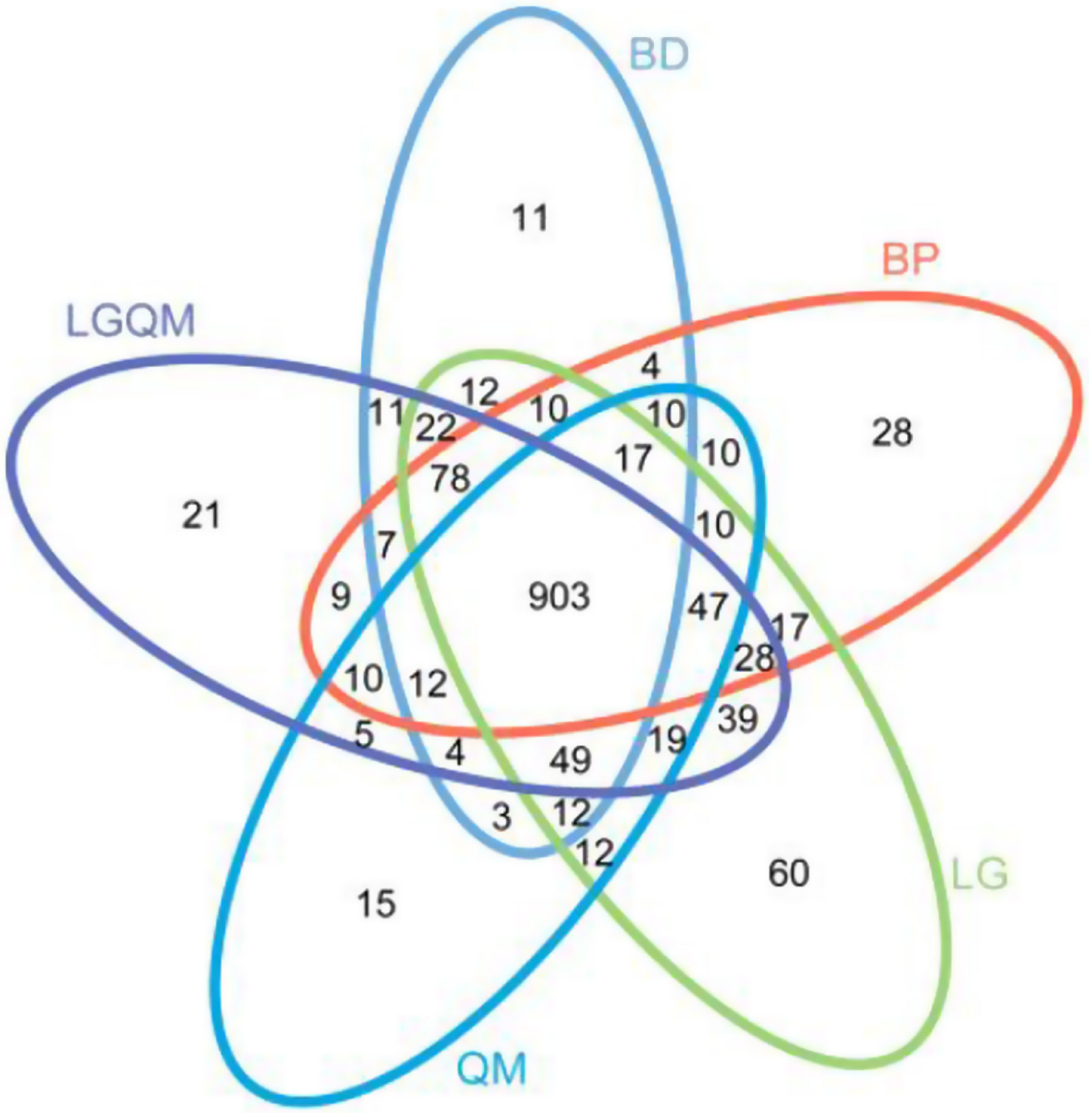
Venn diagram of the soil bacterial community in the five forest vegetation types. BD, *Betula dahurica* forest; BP, *Betula platyphylla* forest; LG, *Larix gmelinii* forest; LGQM, *Q. mongolica* and *L. gmelinii* mixed forest; QM, *Quercus mongolica* forest.

The total number of OTUs of soil bacteria shared among the five forests was 903 (Figure 2), accounting for approximately 60% of all OTUs. The LG forest exhibited the largest number of unique OTUs (60; Figure 2), accounting for 4% of the total OTUs. The number of shared soil bacterial OTUs was the largest for the pair QM and BP (1019; Figure 2), accounting for approximately 68% of the total OTUs.

**FIGURE A3:**
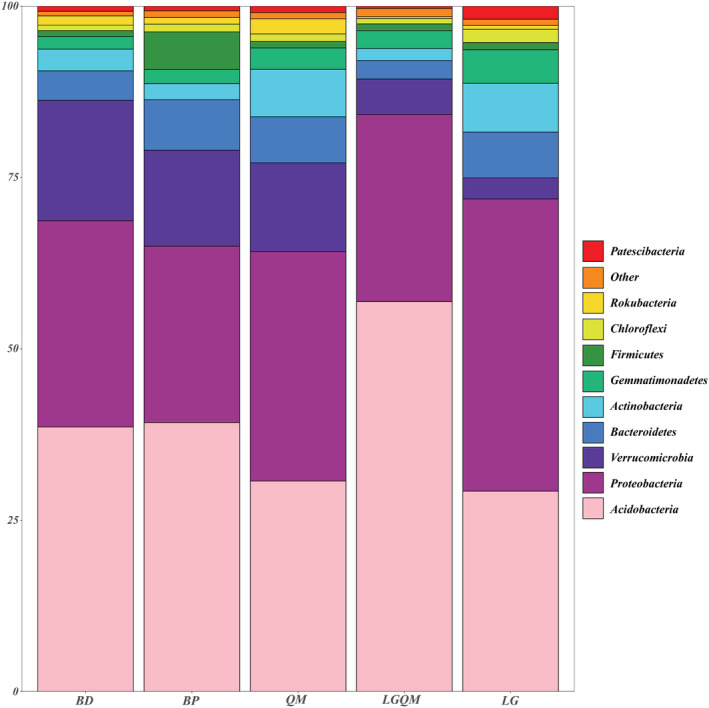
Composition of the top 10 most abundant bacterial phyla in the five forests. BD, *Betula dahurica* forest; BP, *Betula platyphylla* forest; LG, *Larix gmelinii* forest; LGQM, *Q. mongolica* and *L. gmelinii* mixed forest; QM, *Quercus mongolica* forest.

**FIGURE A4:**
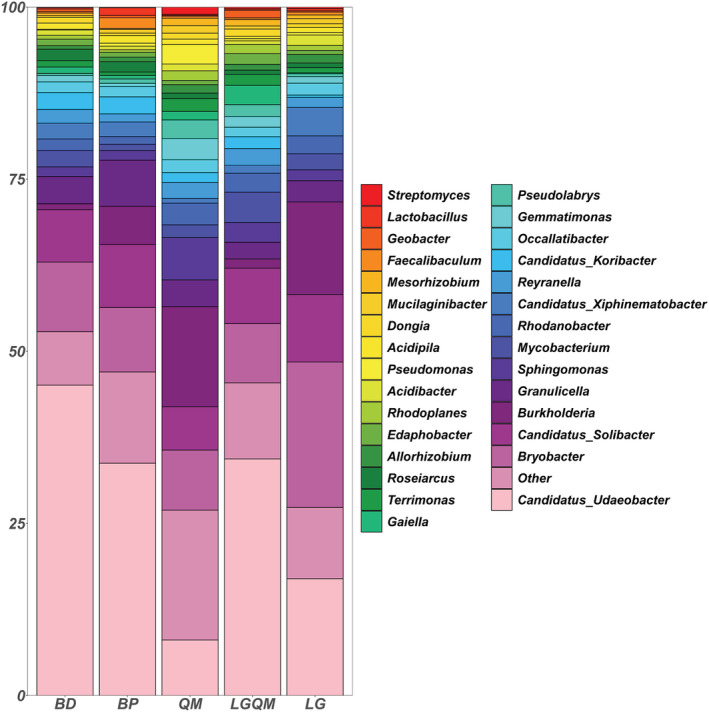
Composition of the most abundant bacterial genera in the five forests. BD, *Betula dahurica* forest; BP, *Betula platyphylla* forest; LG, *Larix gmelinii* forest; LGQM, *Q. mongolica* and *L. gmelinii* mixed forest; QM, *Quercus mongolica* forest.

The relative abundances of bacterial phyla and genera differed among the forest types (Tables A2 and A3 and Figures A3 and A4 in Appendix). Across all samples, 26 bacterial phyla were detected, nine of which had an average abundance >1% (Figure A3 in Appendix). Among them, Acidobacteria was the most abundant phylum, followed by Proteobacteria and Verrucomicrobia (Table A2 and Figure A3 in Appendix). All the soil bacterial phyla differed significantly among forests (Table A2 in Appendix). The relative abundance of Acidobacteria was highest in LGQM (30.7%) and lowest in LG (29.2%), whereas the relative abundance of Proteobacteria was highest in LG (42.6%) and lowest in LG (27.3%). Verrucomicrobia was more abundant in broadleaved forests [BD (17.6%), BP (14.0%), and QM (13.0%)] and coniferous–broadleaved forest [LGQM (5.2%)] than in coniferous forest [LG (3.1%)]. The members of the phylum Firmicutes (5.5%) were dominant in BP but were rare in the other forests.

**FIGURE 3:**
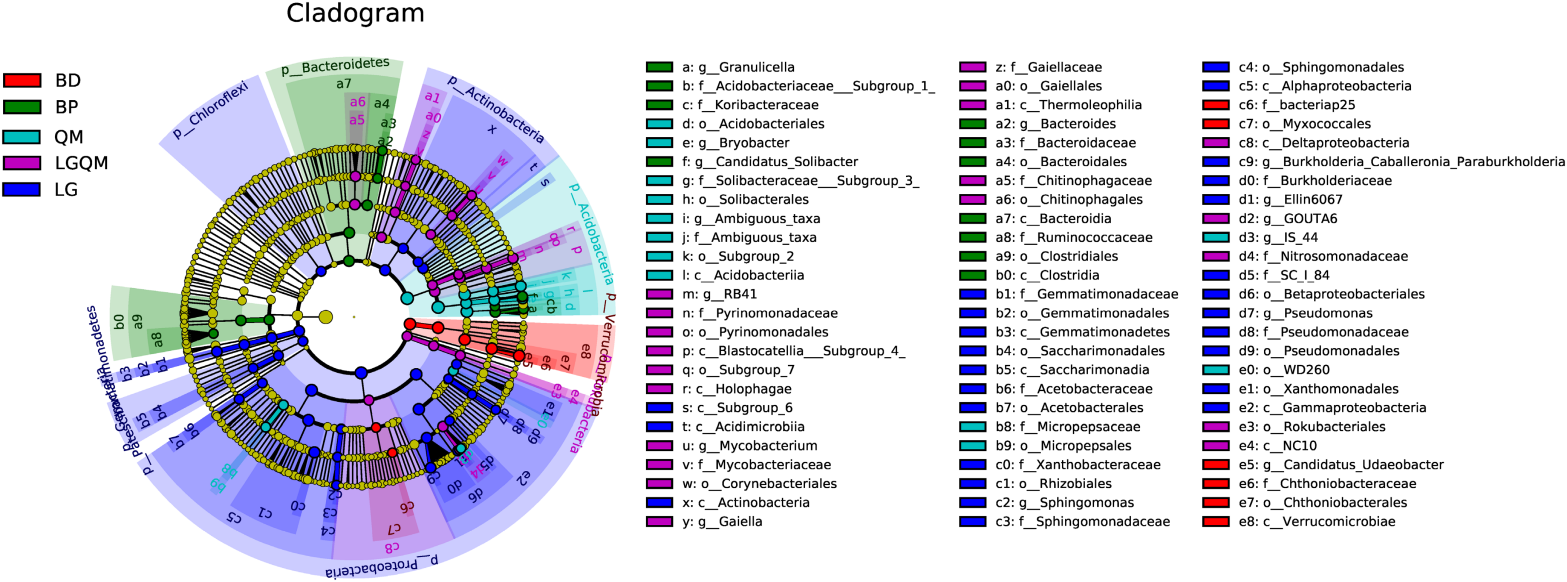
Cladogram of the soil bacterial communities in the five vegetation types of forest soils with liner discriminant analysis (LDA) >3.0. The circles represent bacterial taxa from phyla to genera starting from the center. BD, *Betula dahurica* forest; BP, *Betula platyphylla* forest; LG, *Larix gmelinii* forest; LGQM, *Q. mongolica* and *L. gmelinii* mixed forest; QM, *Quercus mongolica* forest.

The relative abundances of several genera differed significantly between the forests (Table A3 and Figure A4 in Appendix). A total of 69 genera were identified in the five forests (Figure 3), as indicated by liner discriminant analysis (LDA) effect size scores >3.0. At the genus level, *Candidatus_Udaeobacter* was an indicator genus in BD; *Granulicella*, *Candidatus*_*Solibacter*, and *Bacteroides* were indicator genera in BP; *Sphingomonas* and *Pseudomonas* were indicator genera in LG; *Mycobacterium* and *Gaiella* were indicator genera in LGQM; and the indicator genus in QM was ambiguous.

**FIGURE 4:**
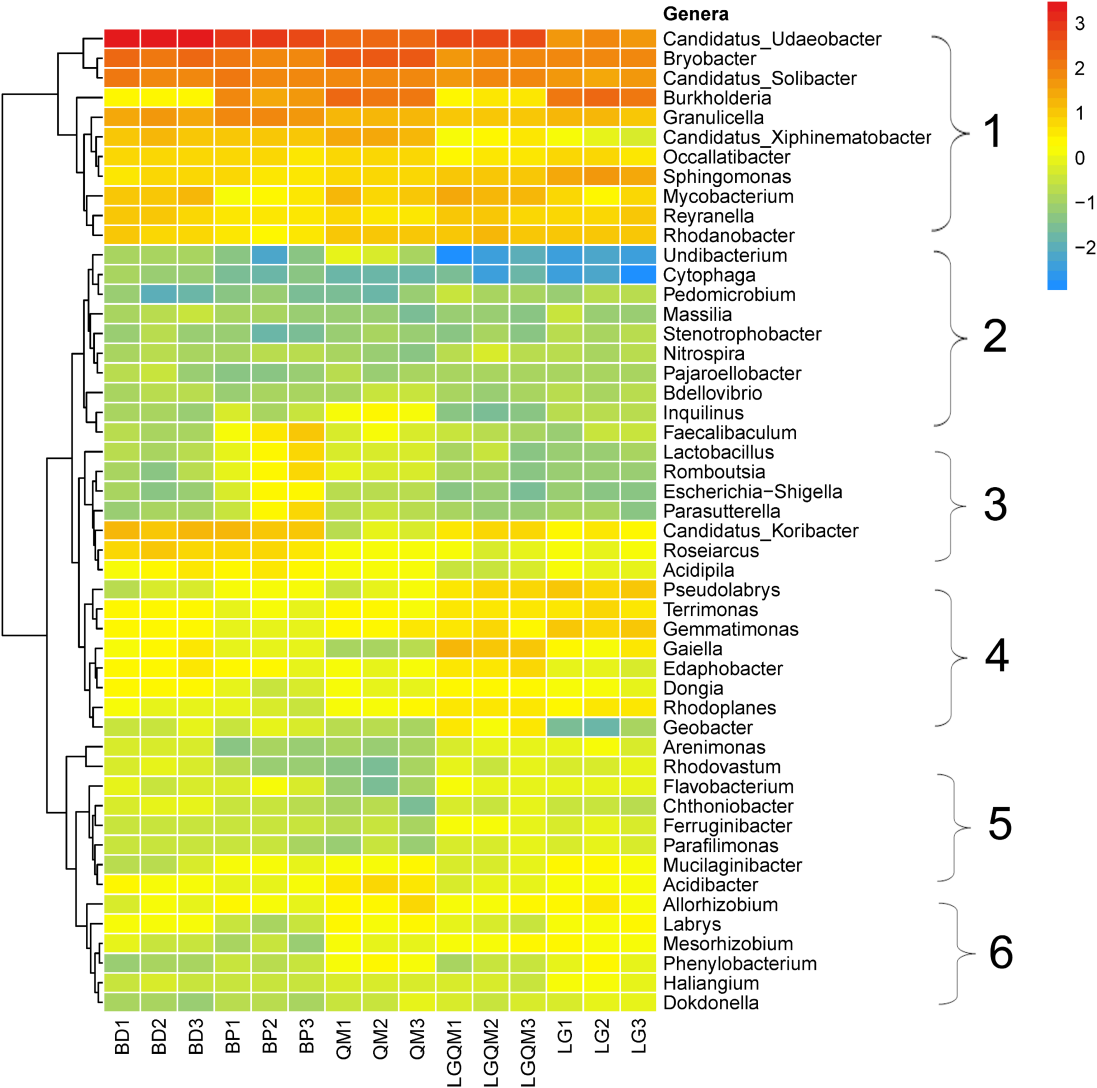
Heatmap based on the relative abundances of the top 50 genera. Red to blue color represents abundance from highest to lowest. BD, *Betula dahurica* forest; BP, *Betula platyphylla* forest; LG, *Larix gmelinii* forest; LGQM, *Q. mongolica* and *L. gmelinii* mixed forest; QM, *Quercus mongolica* forest.

The relative abundances of the top 50 soil bacterial genera were significantly different in the five forests and could be divided into six clusters (Figure 4). In Cluster 1, the four bacterial genera with the highest relative abundances, i.e., *Bryobacter*, *Candidatus_Udaeobacter*, *Burkholderia*, and *Granulicella*, were found in BP, BD, and QM. The five bacterial genera with the highest relative abundances in Cluster 2, i.e., *Faecalibaculum*, *Lactobacillus*, *Romboutsia*, *Escherichia*, and *Parasutterella*, were mainly found in BP. The bacterial genus with the highest relative abundance in Cluster 3, i.e., *Inquilinus*, mostly occurred in the BP forest. The four genera with the highest relative abundances in Cluster 4, i.e., *Pseudolabrys*, *Terrimonas*, *Gemmatimonas*, and *Gaiella*, were primarily found in LGQM and LG forests. The genus with the highest relative abundance in Cluster 5, i.e., *Acidibacter*, was mainly primarily found in the QM forest. The two genera with the highest relative abundances in Cluster 6, i.e., *Mesorhizobium* and *Haliangium*, mostly occurred in the LG forest.

### 3.5 Effect of soil physicochemical properties on soil bacteria

**FIGURE 5:**
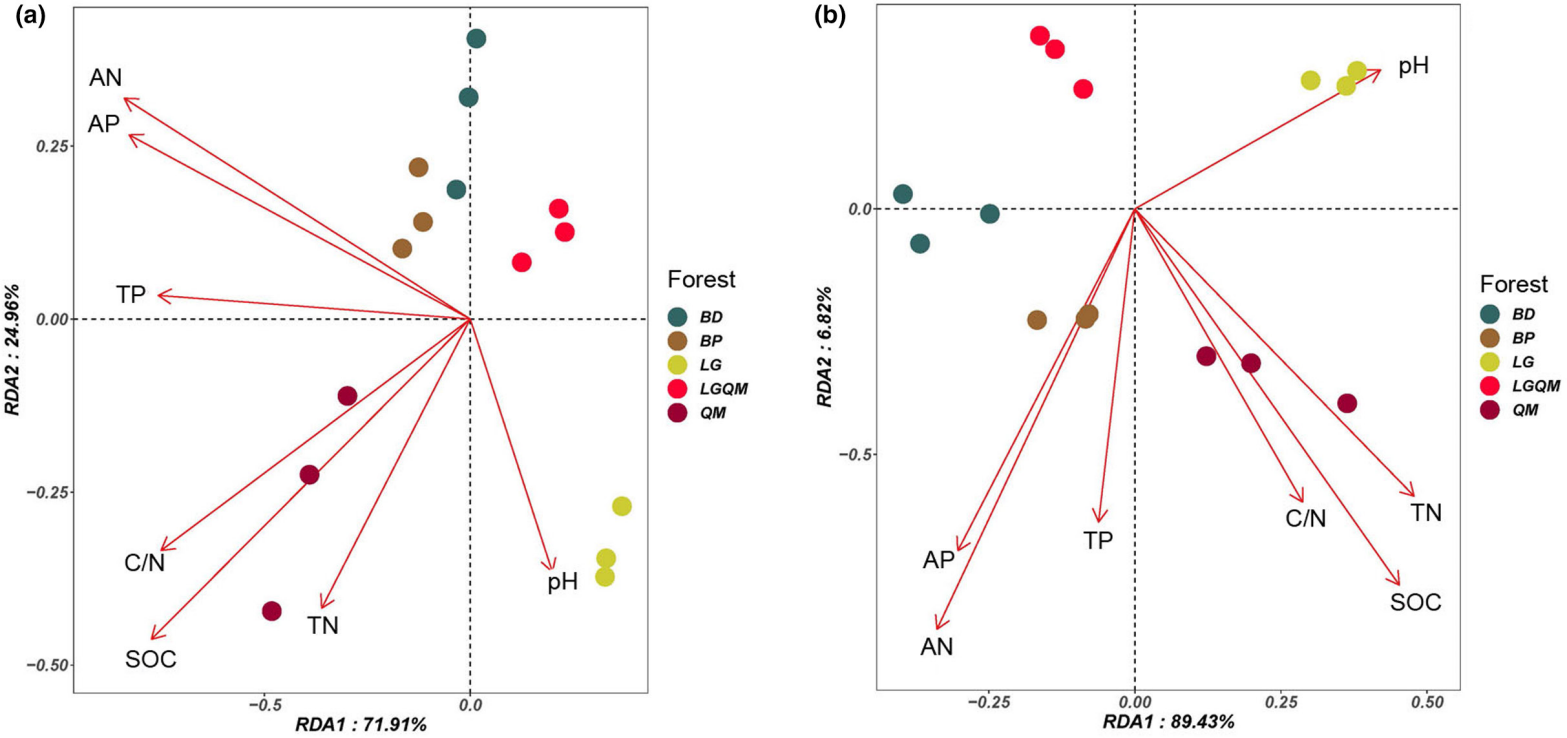
Redundancy analysis (RDA) of dominant soil bacteria phyla (a) and genera (b) constrained by soil physicochemical properties. AN, available nitrogen; AP, available phosphorous; BD, *Betula dahurica* forest; BP, *Betula platyphylla* forest; LG, *Larix gmelinii* forest; LGQM, *Q. mongolica* and *L. gmelinii* mixed forest; QM, *Quercus mongolica* forest; SOC, soil organic carbon; TN, total nitrogen; TP, total phosphorous.

Redundancy analysis indicated that the soil bacterial community structure was significantly correlated with soil physicochemical properties (Figure 5, Table A4 in Appendix). At the phylum level (Figure 5a, Table A4 in Appendix), SOC, C/N, TP, AN, and AP were significantly associated with axis 1 and axis 2. At the genus level (Figure 5b, Table A4 in Appendix), SOC, TN, C/N, TP, AN, and AP were significantly associated with axis 1 and axis 2. Taken together, these findings indicate that the bacterial community structures of the five forests were significantly affected by soil SOC, TN, and C/N (Table A4 in Appendix).

**FIGURE 6:**
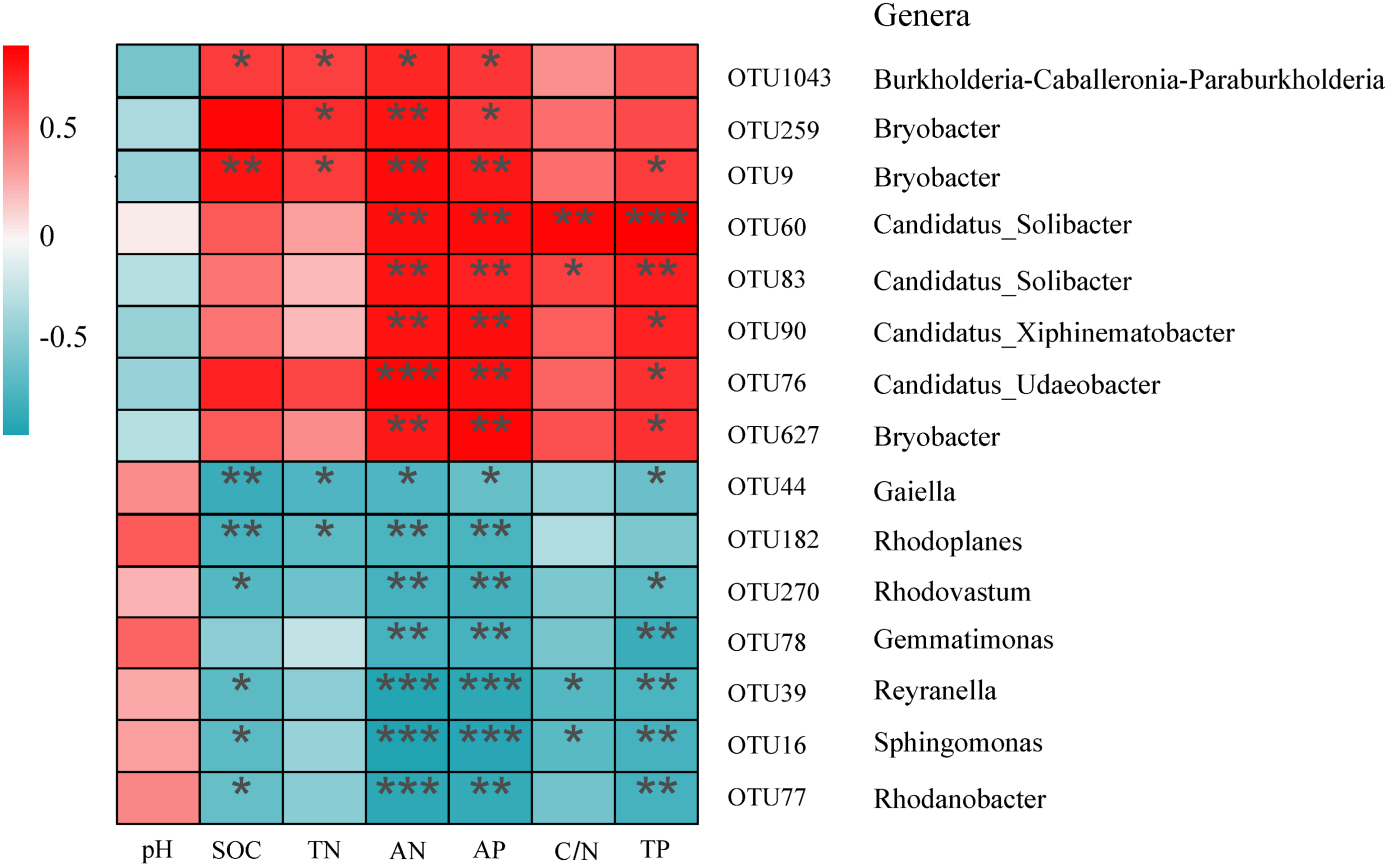
Correlation heatmap of OTUs of bacteria and soil physicochemical properties. Color represents correlation strength, with red for positive and blue for negative correlations. *p < .05, **p < .01, ***p < .001. The heatmap shows the 50 OTUs with the highest correlations with soil physicochemical properties.

Soil physicochemical properties exhibited the strongest effect on bacterial composition and community structure. Therefore, further correlation analyses were conducted between the soil physicochemical properties and the OTUs (Figure 6). A total of 200 bacterial OTUs were found to be significantly (*p* < .05) associated with soil physicochemical properties. Among the top 15 OTUs with the strongest correlation with soil physicochemical properties, excluding the unclassified OTU, eight OTUs were significantly positively and seven OTUs negatively correlated with soil physicochemical properties (e.g., SOC, TN, TP, AP, and C/N; Figure 6). In contrast, no correlation was observed between pH and soil bacteria (Figure 6).

### 3.6 Inferred functionality of soil bacteria and differences between forests

**FIGURE 7:**
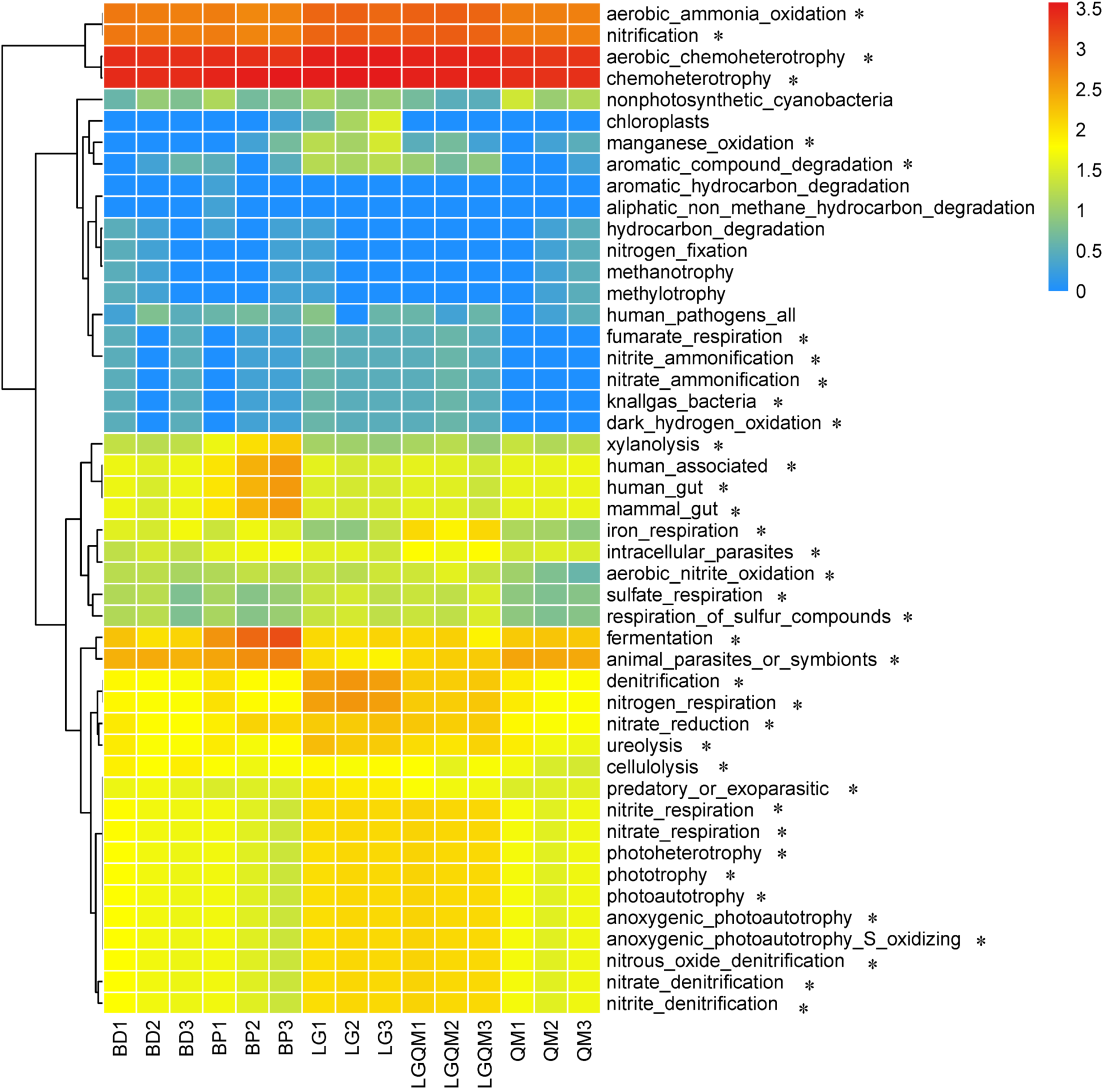
Heatmap of the functional composition of soil bacteria in the five forest vegetation types. The color gradient (red = low, blue = high) represents the relative abundance of the soil bacterial taxa in the different forests. BD, *Betula dahurica* forest; BP, *Betula platyphylla* forest; LG, *Larix gmelinii* forest; LGQM, *Q. mongolica* and *L. gmelinii* mixed forest; QM, *Quercus mongolica* forest. The asterisks indicate significant differences in the relative abundances of bacterial taxa in different forests, according to one‐way ANOVA at a 0.05 significance level.

FAPROTAX analysis detected a total of 47 functional groups (Figure 7). Chemoheterotrophy was the most dominant functional category, followed by nitrification and aerobic ammonia oxidation. Furthermore, the functions differed among the five forests. Phototrophy, nitrate denitrification, nitrite denitrification, nitrate respiration, and nitrite respiration were more prominent in LG and LGQM than in BD, BP, and QM (*p* < .05; Figure 7). In contrast, animal parasites and symbionts and fermentation were more prominent in BD, BP, and QM than in LG and LGQM (*p* < .05; Figure 7).

## 4 DISCUSSION

### 4.1 Effects of forests on soil bacterial diversity

The soil bacterial α diversity in the LG forest was higher than that in the broadleaved forests and mixed coniferous and broadleaved forest. This may be because the LG forest is a climax community with high‐ecosystem stability. During deforestation, the soil habitat is destroyed and the diversity of the soil bacteria decreases. Although secondary succession occurs, the soil ecosystem habitat does not recover, which may be responsible for the decreases in bacterial diversity. Our results were consistent with those from previous studies. For example, Jiang et al. (2021) conducted research on soil bacterial communities in a *Larix gmelinii* forest, as well as in *Pinus sylvestris* and *Betula dahurica* forests, and found that the bacterial α diversity values of the *Larix gmelinii* forest and *Pinus sylvestris* forest were significantly higher than that of the *Betula dahurica* forest. Liu, Wang, et al. (2019) investigated seven different forests in the Daxing'anling region and found that the soil bacterial α diversity of *Pinus koraiensis* forests was significantly higher than that of other forests. In general, the litter decomposition rate in broadleaved forests is higher than that in coniferous forests, thus providing more metabolic substrates for bacterial growth and reproduction, which in turn results in higher biodiversity (Wang et al., 2018). However, these reports are inconsistent with the findings from our study. Zhang et al. (2017) proposed that soil pH can change the nutrient utilization efficiency, physiological metabolic activity, and competition between populations of bacteria, thus directly or indirectly affecting soil bacterial diversity. Therefore, an optimal soil pH range can promote microbial growth. In our study, the soil pH was lowest in the *Betula* forests, suggesting that the higher concentration of hydrogen ions inhibited the proliferation of bacteria (Zheng et al., 2019). In addition, the differences in the composition of aboveground vegetation in our study led to differences in the growth and metabolism of plant roots and the composition and quality of aboveground litter. In turn, this changed the content and physicochemical properties of organic nutrients in the soil, resulting in variations in the soil microorganisms (Deng, Zhang, et al., 2019; Sui et al., 2021). According to the comprehensive NMDS analysis in our study, coniferous forest (LG) and mixed forest (LGQM) belonged to one category, whereas broadleaved forests (BD and BP) belonged to another (Figure 1). The α diversity of soil bacteria in the coniferous forest was higher than in the broadleaved forests and broadleaved and coniferous mixed forests, which may be due to the relationship between soil bacterial diversity and forest age and developmental stage (Sui et al., 2021). *Larix gmelinii* constitutes the original and current dominant community in the study area and its ecological stability is high, whereas other forests were mainly formed after logging activities. Therefore, the soil of the non‐climax forests is easily disturbed by external forces (i.e., the soil stability is low), resulting in low soil bacterial diversity. However, our results are contrary to the intermediate disturbance hypothesis that disturbance increases diversity. This is because the original LG forest belongs to the climax community, with high‐ecosystem stability, resulting in a high diversity of soil bacteria. Secondary forests grow as part of secondary succession following disturbance by humans and soil habitat destruction, and soil bacteria are extremely sensitive to changes in habitat. Moreover, this study area is characterized by a cold temperate climate, meaning that the habitat is difficult to restore once the forest is destroyed, thus also leading to a decrease in soil bacterial diversity. This also shows that climax ecosystems in cold regions have high‐ecosystem functionality that is reduced and difficult to restore after “a persistent decrease”.

### 4.2 Changes in soil bacterial composition in different forests

Soil microorganisms play a critical role in the conversion of organic matter and in biogeochemical cycles (Frey et al., 2020; Hartman et al., 2008), and they have important impacts on forest ecosystems (Deng, Zhou, et al., 2019), plant growth (Li et al., 2014), soil structure (Griffiths et al., 2008), and soil fertility (Yao et al., 2000). Our study identified significant differences between the bacterial community composition of broadleaved forests, mixed coniferous and broadleaved forests, and coniferous forests. Our findings are consistent with those from previous studies indicating that the soil bacterial community composition in hardwood forests differs from that in coniferous forests (Cong et al., 2015; Deng, Zhang, et al., 2019; Wei et al., 2018). This pattern is likely due to differences in soil characteristics resulting from variations in the types of litter and root exudates in each site (Li et al., 2018).

Similar to soil bacterial diversity, the relative abundances of dominant bacterial phyla were also affected by five type in our study. The bacterial community composition of all detected phyla was similar despite the different vegetation types in the studied forests. We found that Acidobacteria and Proteobacteria were the most abundant phyla. Proteobacteria and Acidobacteria are often used as indicators of soil nutrient status (Hartman et al., 2008), and many studies have demonstrated that the relative abundance of Proteobacteria is positively correlated with soil carbon content (Deng, Zhang, et al., 2019; Fierer et al., 2007). Further, Acidobacteria has been reported to be positively correlated with soil pH (Deng, Zhou, et al., 2019; Yang et al., 2018). However, we did not find similar correlations, which may be caused by the limited spatial scale in the present study, with smaller variations in SOC and soil pH.

Acidobacteria are generally oligotrophic (Yang et al., 2019) and play an important role in carbon and nitrogen cycling (Li et al., 2018). The most abundant members of the phylum Acidobacteria are acidophilic, and their relative abundance is therefore significantly higher under acidic conditions (Kalam et al., 2020). In this study, the soil pH ranged from 4.6 to 5.9, explaining why soil acidobacteria were most abundant in all forest soils. Similar results have been reported in temperate forests in northern China (Deng, Zhou, et al., 2019; Yang et al., 2018).

We found that soil nutrient characteristics (e.g., SOC, C/N, AN, TP, AP) were the key environmental factors affecting the soil bacterial community composition (Table A4 in Appendix). Soil nutrient status significantly affects microbial community structure (Xue et al., 2018). The soil C/N ratio can reflect the nutrient balance and is a sensitive indicator of soil quality (Wu et al., 2021). A lower C/N ratio can accelerate the decomposition of microorganisms and the rate of nitrogen mineralization (Högberg et al., 2007). In our study, SOC, C/N, AN, TP, and AP changed significantly with forest type, which led to significant changes in the composition of soil bacteria among the five forest types.

In our study the relative abundance of soil Actinobacteria in the different forests was related to SOC, TN, and AN, which is consistent with results from previous studies (Liu et al., 2020; Liu, Chen, et al., 2019). Actinobacteria has been reported to be one of the most important decomposers of organic matter in soil (Jayasinghe & Parkinson, 2008; Rime et al., 2016). In particular, Mitra et al. (2008) found that Actinobacteria in secondary forests had a significant positive correlation with SOC in the process of vegetation succession. Soil nutrient characteristics were clearly key factors affecting the soil bacterial communities in the different forests considered here.

FAPROTAX is an effective tool for predicting soil bacterial function (Louca et al., 2016). In this study, the FAPROTAX method indicated that some bacterial functional groups (e.g., aerobic ammonia oxidation, chemoheterotrophy, nitrification) differed significantly among the five forests (Figure 7). This finding indicates that changes in the bacterial composition would influence soil Carbon and nutrient cycling functions (Frey et al., 2016). Moreover, in our study phototrophy, nitrate denitrification, nitrite denitrification, nitrate respiration, and nitrite respiration had crucial functions in soil carbon and nitrogen cycling in coniferous forests and mixed coniferous and broadleaved forests, while parasites and symbionts and fermentation were found to be more important in broadleaved forests. Vegetation changes often alter soil physicochemical properties, making broadleaved forests different compared with coniferous forests. Therefore, because of differences in soil nutrient contents, the soil bacterial functions may have differed significantly between the five forests.

## 5 CONCLUSION

Inconsistent with the intermediate disturbance hypothesis, our results showed that the soil bacterial diversity in a climax forest was significantly higher than in degraded forests, which does not support our second hypothesis. The soil nutrient contents, however, did not show consistent patterns among the five forest types, leading to the rejection of our first hypothesis. Our results indicate that disturbances and forest degradations will result in changes in soil physicochemical properties, which will further affect the diversity, community composition, and functions of soil bacteria, ultimately influencing forest ecosystem functioning and services.

## AUTHOR CONTRIBUTIONS

**Mengsha Li:** Writing – original draft (equal); writing – review and editing (equal). **Beat Frey:** Writing – review and editing (equal). **Mingyu Wang:** Data curation (equal). **Xiaohong Weng:** Investigation (equal). **Xin Wang:** Methodology (equal); resources (equal). **Fuyuan Chen:** Investigation (equal). **Xianda Li:** Investigation (equal). **Zhong Du:** Methodology (equal); visualization (equal). **Libin Yang:** Investigation (equal); methodology (equal); visualization (equal); writing – review and editing (equal). **Xin Sui:** Funding acquisition (lead); methodology (lead); software (lead); supervision (lead); validation (lead); visualization (lead); writing – original draft (lead). **Mai‐He Li:** Writing – review and editing (equal).

## FUNDING INFORMATION

This work was funded by the Heilongjiang Province Postdoctoral Research Start‐up Fund Project (LBH‐Q21167); the Natural Sciences Foundation of Heilongjiang Province (LH2020C088); the Outstanding Youth Foundation of Heilongjiang University (JCL202006); and the China Scholarship Council Visiting Scholar Program (201908230401).

## CONFLICT OF INTEREST

The authors declare that the research was conducted in the absence of any commercial or financial relationships that could be construed as a potential conflict of interest.

## Data Availability

Raw data of bacterial sequences were deposited into the NCBI Sequence Read Archive under accession number PRJNA691134.

## Appendix

**TABLE A1:** Characteristics of the climax forest and the four degraded forests in the Heilongjiang Zhongyangzhan Black‐billed Capercaillie Nature Reserve.

Vegetation type	Primary tree species	Elevation (m a.s.l.)	Forest type	Litter (kg·hm^−2^·a^−1^)	Shannon diversity
BD	*Betula dahurica*, *Populus tremula*	552.1	Secondary forest	5983	1.55
BP	*Betula platyphylla*, *Populus tremula*	535.5	Secondary forest	5680	1.23
QM	*Quercus mongolica*, *Tilia amurensis*	520.4	Secondary forest	5520	1.53
LGQM	*Larix gmelinii*, *Betula platyphylla*, *Populus tremula*, *Quercus mongolica*	565.4	Secondary forest	7297	1.67
LG	*Larix gmelinii*	585.7	Climax forest	9230	0.58

Abbreviations: BD, *Betula dahurica*; BP, *Betula platyphylla*; LG, *Larix gmelinii*; LGQM, *Q. mongolica* and *L. gmelinii* mixed forest; QM, *Quercus mongolica*.

**TABLE A2:** Relative abundances of the most abundant bacterial phyla (>1%) in the five forests.

Forest	Acidobacteria	Proteobacteria	Verrucomicrobia	Bacteroidetes	Actinobacteria
BD	9773.3 ± 173.9 b	7616.0 ± 193.6 c	4445.7 ± 274.3 a	1083.7 ± 112.8 b	803.7 ± 15.3 b
BP	9935.0 ± 954.7 b	6515.7 ± 421.5 d	3540.7 ± 252.3 b	1859.3 ± 495.7 a	593.7 ± 51.8 b
QM	14397.7 ± 398.3 a	6911.7 ± 394.1 d	1307.0 ± 47.3 c	685.7 ± 21.6 b	448.0 ± 55.1 b
LGQM	7778.3 ± 547.4 c	8463.7 ± 199.6 b	3281.7 ± 346.0 b	1703.7 ± 60.6 a	1750.7 ± 308.8 a
LG	7402.7 ± 220.0 c	10789.0 ± 283.5 a	778.3 ± 86.0 d	1687.7 ± 187.1 a	1798.7 ± 306.1 a
**Forest**	**Gemmatimonadetes**	**Firmicutes**	**Chloroflexi**	**Rokubacteria**	
BD	471.7 ± 25.1 d	218.0 ± 56.0 b	199.0 ± 11.3 c	340.7 ± 13.9 b	
BP	523.3 ± 66.7 cd	1393.0 ± 801.0 a	290.0 ± 24.6 b	240.7 ± 52.6 c	
QM	655.0 ± 50.1 bc	255.3 ± 32.6 b	196.0 ± 14.7 c	67.7 ± 8.4 e	
LGQM	787.0 ± 97.6 b	240.3 ± 47.4 b	281.0 ± 13.5 b	560.3 ± 50.5 a	
LG	1243.0 ± 146.0 a	271.0 ± 73.7 b	485.3 ± 51.6 a	141.0 ± 17.1 d	

*Note*: All values are reported as mean ± standard deviation (*n* = 3). Different letters within a row indicate significant differences (*p* < .05).

Abbreviations: BD, *Betula dahurica* forest; BP, *Betula platyphylla* forest; LG, *Larix gmelinii* forest; LGQM, *Q. mongolica* and *L. gmelinii* mixed forest; QM, *Quercus mongolica* forest.

**TABLE A3:** Relative abundances of the most abundant bacterial genera (top 10) in the five forest soils.

Forest	*Candidatus_Udaeobacter*	*Bryobacter*	*Candidatus_Solibacter*	*Granulicella*	*Candidatus_Koribacter*
BD	4159.7 ± 263.3 a	934.0 ± 86.8 b	701.0 ± 23.5 b	366.7 ± 46.5 b	228.0 ± 10.8 a
BP	3276.7 ± 253.5 b	909.0 ± 103.7 b	889.0 ± 88.0 a	648.0 ± 99.5 a	240.0 ± 26.9 a
QM	1022.7 ± 54.4 c	1272.7 ± 77.1 a	590.7 ± 13.6 c	185.7 ± 20.3 c	20.7 ± 7.1 d
LGQM	3107.7 ± 322.5 b	778.3 ± 118.0 bc	727.0 ± 58.9 b	218.0 ± 9.5 c	156.3 ± 3.1 b
LG	668.3 ± 79.7 c	720.3 ± 9.9 c	521.0 ± 18.7 c	321.0 ± 43.6 b	118.3 ± 8.5 c

Forest	*Mycobacterium*	*Candidatus_Xiphinematobacter*	*Reyranella*	*Rhodanobacter*	*Roseiarcus*
BD	219.3 ± 6.7 b	211.7 ± 24.0 b	183.3 ± 25.7 b	154.0 ± 26.1 b	152.0 ± 21.5 a
BP	87.7 ± 28.4 c	205.3 ± 5.5 b	113.7 ± 7.6 c	110.0 ± 9.0 c	147.3 ± 28.6 a
QM	139.7 ± 41.0 bc	248.7 ± 12.0 a	87.7 ± 12.4 c	157.7 ± 16.2 b	41.3 ± 6.7 b
LGQM	397.0 ± 96.6 a	105.0 ± 20.1 c	219.0 ± 14.7 a	248.7 ± 27.6 a	56.0 ± 18.5 b
LG	152.7 ± 54.9 bc	58.0 ± 16.7 d	192.0 ± 21.7 ab	261.0 ± 14.5 a	65.7 ± 15.5 b

*Note*: All values are reported as mean ± standard deviation (*n* = 3). Different letters within a row indicate significant differences (*p* < .05).

Abbreviations: BD, *Betula dahurica* forest; BP, *Betula platyphylla* forest; LG, *Larix gmelinii* forest; LGQM, *Q. mongolica* and *L. gmelinii* mixed forest; QM, *Quercus mongolica* forest.

**TABLE A4:** Significance of the soil physicochemical properties in explaining the community structure obtained from the redundancy analysis (RDA) results.

	Phyla		Genera	
	*r* ^2^	*p*	*r* ^2^	*p*
pH	.19	>.05	.29	>.05
SOC	.85	<.01	.88	<.01
TN	.31	>.05	.63	<.01
C/N	.71	<.01	.49	<.05
TP	.64	<.01	.47	<.05
AN	.92	<.01	.96	<.01
AP	.86	<.01	.66	<.01

Abbreviations: AN, available nitrogen; AP, available phosphorous; SOC, soil organic carbon; TN, total nitrogen; TP, total phosphorous.
